# Do older people with cardiovascular-metabolic multimorbidity prefer to sign contracts for family doctor services? Evidence from a cross-sectional study in rural Shandong, China

**DOI:** 10.1186/s12877-021-02543-w

**Published:** 2021-10-20

**Authors:** Shijun Yang, Jie Li, Peipei Fu, Yan Chen, Yi Wang, Dan Zhao, Zhixian Li, Wenjuan Li, Jie Li, Chen Yan, Zhen Gui, Chengchao Zhou

**Affiliations:** 1 Centre for Health Management and Policy Research, School of Public Health, Cheeloo College of Medicine, Shandong University, Jinan, 250012 China; 2 School of Public Health, Wannan Medical College, Wuhu, 241002 China; 3 NHC Key Lab of Health Economics and Policy Research, Shandong University, 44 Wen-hua-xi Road, Jinan, 250012 China

**Keywords:** Cardiovascular-metabolic multimorbidity, Rural older people, Family doctor contract services

## Abstract

**Background:**

Family doctor policy is an important part of deepening healthcare reform in China. The study aimed to explore the association between cardiovascular-metabolic multimorbidity and the status of signing a contract for family doctor services among the older people in rural Shandong, China.

**Methods:**

A cross-sectional study was conducted in 3 cities of Shandong province, China. A total of 1395 rural residents over 60 years of age were included in this study using a multistage stratified random sampling method. Covariates included demographic and socioeconomic characteristics, health-related characteristics, health service utilization, and awareness of family doctor contract services. The univariate and multivariate regression logistic analysis was used to analyze the data.

**Results:**

There were 28.2% of the rural older people contracted for the family doctor contract services. The contract rate of seniors with cardiovascular-metabolic multimorbidity was statistically higher than those without cardiovascular-metabolic multimorbidity (OR = 1.67, 95%CI, 1.21-2.32) after controlling for confounding factors. In addition, occupation, physical activities, self-rated health status, distance from the village clinic, the awareness of family doctor contract services were found to be associated with the signing behavior among the rural older adults.

**Conclusion:**

This study demonstrated that the rural older people with cardiovascular-metabolic multimorbidity had a higher family doctor contract rate than those without cardiovascular-metabolic multimorbidity, and there was a gap between the current signing rate and the policy goal. To increase the rate of signing for family doctor contract services, the government should take joint efforts to expand the publicity and coverage, and give priority to meeting the healthcare demands of rural older adults with cardiovascular-metabolic multimorbidity.

## Background

Family doctor contract services (FDCSs) policy was adopted to provide healthcare services and health management for the whole population by signing agreements with residents’ families [1]. It is based on the principles of complete notification, voluntary contracting, and standardized services. In China, family doctor team provide the basic healthcare services to those residents who have signed FDCSs contract. The basic service package is free. With the upgrade of the service package and the situation in different regions, there would be various standards of contracted service fee [2]. FDCSs policy has been proven to be effective in improving health status of residents [3, 4]. In 2016, FDCSs policy was carried out widely in China, especially for those targeting key subgroups. The older people and residents with chronic diseases are the main priority subgroups in contracting for family doctor services [5]. The doctor team in rural area serves as the “gatekeeper” of residents’ health and the executor of FDCSs policy, the key groups are further targeted and managed by them [5].

By 2050, China’s total older population will exceed 400 million and the proportion of people over 60 years old will exceed 30% [6]. In this study, we defined the seniors as people who were 60 years old and above. Several studies have revealed that advanced age was associated with a higher signing rate for family doctor services [7–9]. A preliminary study in Shanghai found that advanced age was an important factor to increase the contract rate [7]. Another study found that older adults (especially frail older people) were more likely to receive in-home (long-term) care by family doctors [9]. As one of the key groups of FDCSs policy, there has been many relevant studies on the contract status among the older people.

Older people are at high risk of chronic diseases [10]. A previous study has shown that each older adult could suffer from 2.55 types of chronic diseases on average and the per capita out-of-pocket medical expenses for chronic diseases was 4532 yuan for half a year, while most of the patients have been suffering from chronic diseases for more than 10 years [11]. Several studies have revealed a correlation between higher signing rates and chronic diseases such as hypertension, diabetes, and cardiovascular diseases [8, 9, 12]. Studies have also demonstrated that FDCSs signing status was associated with better self-management of chronic diseases [13–15]. These findings indicated that the residents with chronic diseases have higher signing rates for better chronic disease management, especially the older adults.

With the aging of the population, more and more older adults were threatened by various chronic diseases [10]. When a person suffers from two or more chronic diseases at the same time, it is called multimorbidity [16–18]. According to previous studies, there were several patterns of multimorbidity [19]. Cardiovascular-metabolic multimorbidity was one of the most common one among those patterns [17, 19]. It was revealed that older age was one of the key factors associated with cardiovascular-metabolic multimorbidity [20]. The prevalence of cardiovascular-metabolic multimorbidity in the older adults was significantly higher than the younger group [17, 21]. A previous study indicated that the prevalence of multimorbidity was even higher among the older people in rural China than their urban counterparts [20]. However, it is still unclear whether there is a correlation between FDCSs signing status and cardiovascular-metabolic multimorbidity among the rural older people.

Therefore, we hypothesized that cardiovascular-metabolic multimorbidity was an important factor affecting the FDCSs contract status of rural older adults. We tested this hypothesis by using the data from the baseline survey of rural FDCSs in Shandong Province, China in 2018. The findings of this study would probably contribute to the development of personalized and targeted FDCSs, and optimize relevant policies.

## Methods

### Design and participants

**Fig. 1:**
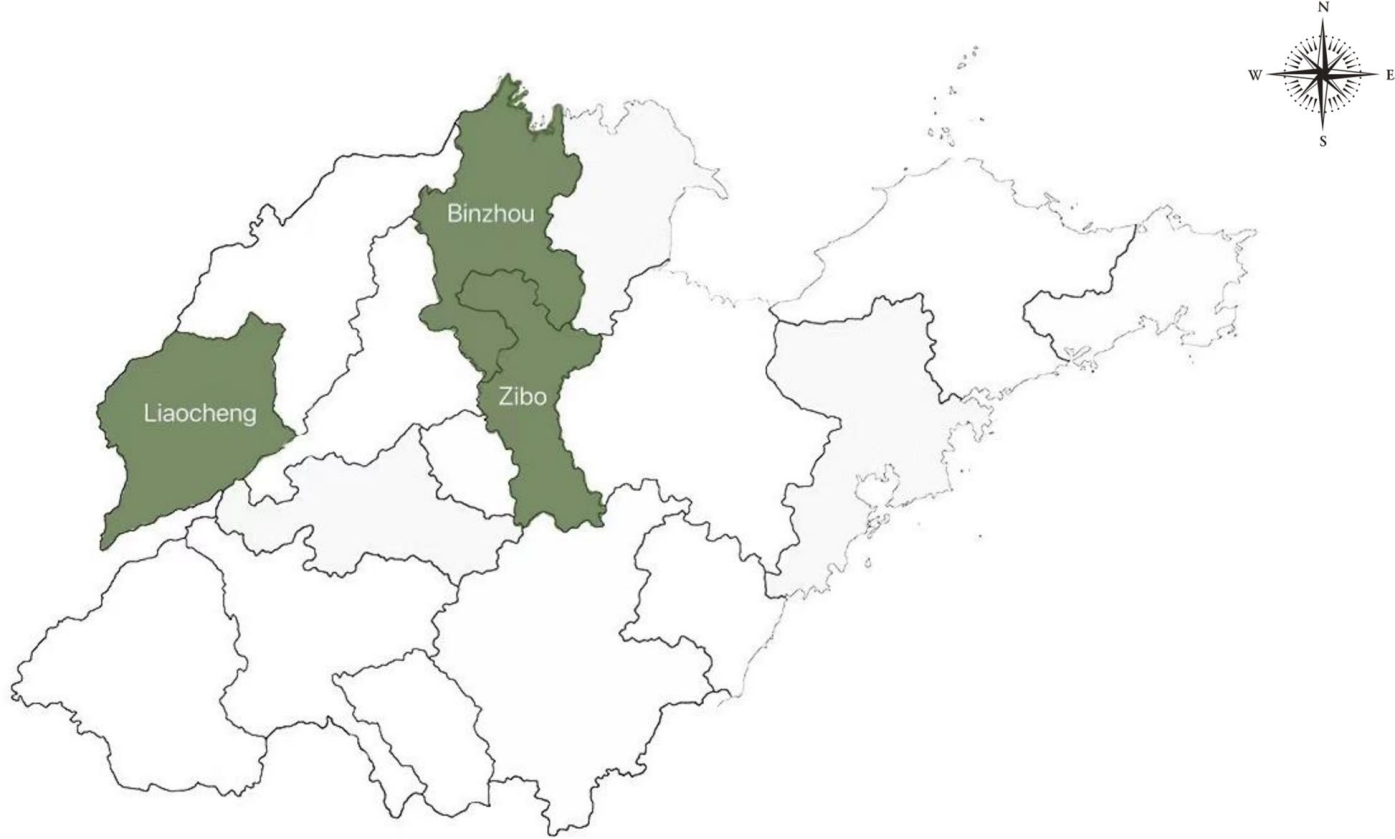
Location of the study sites in Shandong province

A cross-sectional study was conducted in Shandong Province, the second-most populous province in China. A multilevel stratified random sampling method was employed to select 3 cities, including Zibo, Liaocheng, Binzhou (Fig. 1), according to the economic development level, and 2 counties in each city were chosen randomly.

**Fig. 2:**
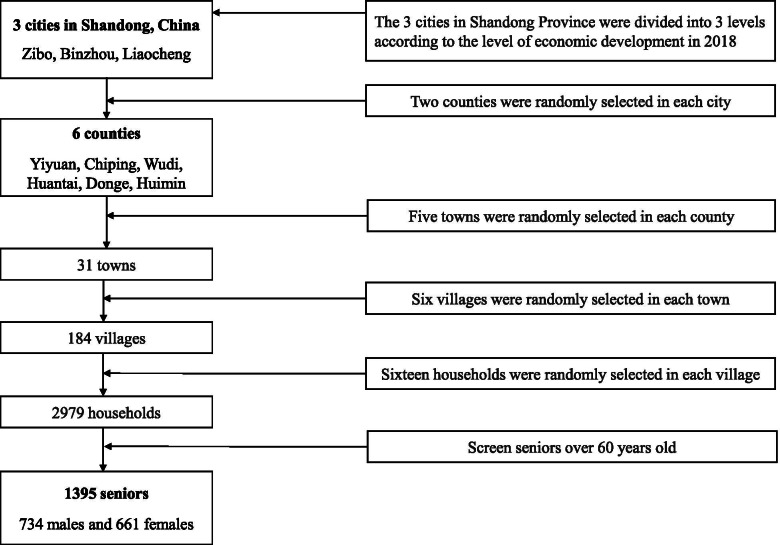
Flowchart of the sampling method

Within each county, 5 townships and 6 villages in each township were randomly chosen, and then 16 households were randomly selected from each village by using random numbers function in Excel. In total, 3000 questionnaires were distributed, and 2979 valid questionnaires were included in this study, with a response rate of 99.3%. Finally, a total of 1395 older respondents over 60 years old (734 males and 661 females) were included in the analysis (Fig. 2).

The research protocol was approved by the Ethical Committee of the School of Public Health, Shandong University. All participants provided written informed consent after a detailed explanation of the research protocol, and we received all the participants’ informed consents.

### Questionnaire

We collected information through a structured questionnaire in Chinese to analyze the FDCS contracting rate and its determinants in rural residents. Based on this purpose, the research group conducted several discussions to develop the questionnaire. A pilot survey was conducted in two villages of Jinan city, and the questionnaire was refined by the research group. The Cronbach’s alpha was over 0.7, which indicates a high reliability of the questionnaire. After that, a face-to-face survey was conducted by well-trained investigators. Completed questionnaires were carefully checked by the supervisors after the interview each day.

### Variables and measurement

Cardiovascular-metabolic multimorbidity was defined as the co-occurrence of two or more chronic diseases (including hypertension, diabetes and coronary heart disease) within the same individual in our study. Signing status was measured by the question “Did you contract with the family doctor this year?”. Respondents answer “yes” or “no” for this question. Covariates included demographic characteristics (age, gender, educational attainment, marital status, empty nest), socioeconomic characteristics (occupation, household income, catastrophic health expenditure (CHE), city), health service utilization (distance from village clinic), awareness of FDCSs, and health-related characteristic (hypertension, diabetes, coronary heart disease, cardiovascular-metabolic multimorbidity, smoking status, drinking status, physical activities, self-rated health status) [22, 23]. Older adults who were empty nest referred to those elderly who did not live with their children (including those older people who lived alone or lived with a spouse) [24]. City included three prefecture-level sampling cities: Zibo, Liaocheng, Binzhou, respectively represented three economic development levels. Rural areas in the above three prefecture-level cities were randomly chosen. The awareness of FDCSs was measured by the question “How well do you know about FDCSs?”. The answer was “know it well” or “less familiar”. Most of the covariates mentioned above have previously been reported to be associated with the status of contracting for family doctor service [11, 25–28].

### Statistical methods

EpiData version 3.1 was used to double enter and encode the data. Stata/SE 15.1 was used to analyze the data. The statistical significance threshold was set at *p* < 0.05 (two-tailed) for all analyses. Frequency and percentage were used to describe the baseline characteristics of the respondents. Then we used the Chi-square tests and t-tests to compare the differences for categorical and continuous variables respectively. Multivariate logistic regression model was conducted to explore the factors associated with FDCSs contract behavior as well as to control the influence of confounding factors. To examine the relationship between cardiovascular-metabolic multimorbidity and contract status of FDCSs, we conducted two models for comparison. Specifically, Model 1 was the unadjusted model which did not control the covariates, and Model 2 was the adjusted model after controlling the covariates.

### Results

Table 1 presents the basic characteristics of the participants. There were 28.2% of older people who signed contracts with their family doctors. Among the 1395 older respondents, the mean age was 68.1 (SD = 5.7) years, with 47.4% female. Majority of the respondents were married (80.4%), with junior education or below (55.3%), did the agricultural job (54.6%), less familiar with FDCSs (89.8%). Table 2 presents the health-related characteristics of the participants. Majority of the respondents were never smokers (60.0%), never drinkers (57.5%), without multimorbidity (68.0%), have physical activities (55.8%), good self-rated health status (36.34%).

**Table 1:** Basic characteristics of the rural older people signed status of FDCSs in Shandong, China, 2018

Variable	N	Percentage (%)	Signed status of family doctor contract services			
			Yes	No	χ^2^/ t-test	*P*-Value
*n* = 1395			393 (28.17)	1002 (71.83)		
**Gender**					12.97	< 0.01
Male	734	52.62	237 (60.31)	497 (49.60)		
Female	661	47.38	156 (39.69)	505 (50.40)		
**Educational attainment**					30.11	< 0.01
Illiteracy	508	36.42	102 (25.95)	406 (40.52)		
Junior education or below	772	55.34	244 (62.09)	528 (52.69)		
Senior high or above	115	8.24	47 (11.96)	68 (6.79)		
**Marital status**					1.51	0.22
Married	1121	80.36	324 (82.44)	797 (79.54)		
Unmarried	274	19.64	69 (17.56)	205 (20.46)		
**Empty nest**					0.69	0.406
Yes	710	50.90	207 (52.67)	503 (50.20)		
No	685	49.10	186 (47.33)	499 (49.80)		
**Occupation**					–	0.251
Unemployed	448	32.11	135 (34.35)	313 (31.24)		
Farming	761	54.55	202 (51.40)	559 (55.79)		
Farming+ nonfarm work	81	5.81	20 (5.09)	61 (6.09)		
Nonfarm work	45	3.23	13 (3.31)	32 (3.19)		
Retirees	60	4.30	23 (3.69)	37 (5.85)		
**Household income**					14.28	0.003
Q1(Poorest)	351	25.16	73 (18.58)	278 (27.74)		
Q2	347	24.87	99 (25.19)	248 (24.75)		
Q3	346	24.81	114 (29.01)	232 (23.15)		
Q4(Richest)	351	25.16	107 (27.23)	244 (24.35)		
**CHE**					1.16	0.281
Yes	692	49.61	204 (51.91)	488 (48.70)		
No	703	50.39	189 (48.09)	514 (51.30)		
**City**					24.39	< 0.01
Liaocheng	513	36.77	163 (41.48)	350 (34.93)		
Binzhou	442	31.68	86 (21.88)	356 (35.53)		
Zibo	440	31.55	144 (36.64)	296 (29.54)		
**Distance from village clinic**					7.63	0.022
< 100 m	322	23.08	74 (18.83)	248 (24.75)		
100-500 m	772	55.34	239 (60.81)	533 (53.19)		
> 500 m	301	21.58	80 (20.36)	221 (22.06)		
**Awareness of FDCSs**					206.45	< 0.01
Less familiar	1253	89.82	280 (71.25)	973 (97.11)		
Know it well	142	10.18	113 (28.75)	29 (2.89)		

FDCSs: family doctor contract services; CHE: catastrophic health expenditure

- Use Fisher’s exact probability method, there is no corresponding statistical value

**Table 2:** Health-related characteristics of rural older people FDCSs signed status in Shandong, China, 2018

Variable	N	Percentage (%)	Signed status of family doctor contract services			
			Yes	No	χ^2^	*P*-Value
n = 1395			393 (28.17)	1002 (71.83)		
**Hypertension**					0.70	0.403
Yes	646	46.31	189 (48.09)	457 (45.61)		
No	749	53.69	204 (51.91)	545 (54.39)		
**Diabetes**					3.20	0.074
Yes	184	13.19	62 (15.78)	122 (12.18)		
No	1211	86.81	331 (84.22)	880 (87.82)		
**Coronary heart disease**					6.73	0.009
Yes	292	20.93	100 (25.45)	192 (19.16)		
No	1103	79.07	293 (74.55)	810 (80.84)		
**Cardiovascular-metabolic multimorbidity**					9.15	0.002
Yes	446	31.97	98 (24.94)	178 (17.76)		
No	949	68.03	295 (75.06)	824 (82.24)		
**Smoking status**					3.30	0.192
Never smokers	837	60.00	221 (56.23)	616 (61.48)		
Former smokers	264	18.92	80 (20.36)	184 (18.36)		
Current smokers	294	21.08	92 (23.41)	202 (20.16)		
**Drinking status**					9.03	0.011
Never drinkers	802	57.49	201 (51.15)	601 (59.98)		
Former drinkers	193	13.84	62 (15.78)	131 (13.07)		
Current drinkers	400	28.67	130 (33.08)	270 (26.95)		
**Physical activities**					21.34	< 0.01
Yes	779	55.84	258 (65.65)	521 (52.00)		
No	616	44.16	135 (34.35)	481 (48.00)		
**Self-rated health**					11.57	0.003
Good	507	36.34	167 (42.49)	340 (33.93)		
Normal	484	34.70	134 (34.10)	350 (34.93)		
Poor	404	28.96	92 (23.41)	312 (31.14)		

*FDCSs* family doctor contract services

Compared to the seniors who had not signed contracts with family doctors, those who had signed were more likely to be: male, with senior high or above education, have higher household income, have two types of chronic diseases or above (cardiovascular-metabolic multimorbidity), have drinking experience (including former drinkers and current drinkers), have physical activities, good self-rated health status, 100-500 m away from village clinic, know more about FDCSs. Liaocheng had the highest singing rate, followed by Binzhou, and Zibo was the lowest (Shown in Tables 1 and 2).

The results of this study were presented to estimate the association between cardiovascular-metabolic multimorbidity and FDCSs signing behavior of the rural older people using two models (Table 3). According to the unadjusted model (Model 1), the contract rate of the older adults with cardiovascular-metabolic multimorbidity was statistically higher than those older adults without cardiovascular-metabolic multimorbidity (OR = 1.54, 95% CI, 1.16 - 2.03). When controlling for other variables (Model 2), the signing rate of the older people with cardiovascular-metabolic multimorbidity was still statistically higher than those without cardiovascular-metabolic multimorbidity (OR = 1.67, 95% CI, 1.21 - 2.32). The Hosmer-Lemeshow Test for the regression showed that Model’s goodness-of-fit was 0.785, which indicated the model fit well.

**Table 3:** Association between FDCSs status of rural older people and cardiovascular-metabolic multimorbidity, Shandong, China 2018

Variable	Model 1 (No covariates)			Model 2 (Covariates)		
	OR	95% CI	*P*	OR	95% CI	*P*
**Cardiovascular-metabolic multimorbidity**						
No	1.0			1.0		
Yes	1.54	1.16 - 2.03	0.003	1.67	1.21 - 2.32	0.002
**Occupation**						
Unemployed				1.0		
Agricultural work				0.79	0.58 - 1.07	0.132
Agricultural work + non-agricultural work				0.49	0.26 - 0.93	0.030
Non-agricultural work				0.49	0.21 - 1.14	0.098
Retirees				0.43	0.19 - 0.93	0.033
**Drinking status**						
Never drinkers				1.0		
Former drinkers				1.10	0.69 - 1.75	0.687
Current drinkers				1.23	0.84 - 1.79	0.291
**Physical activities**						
No				1.0		
Yes				1.34	1.01 - 1.76	0.040
**Self-rated health**						
Good				1.0		
Normal				0.85	0.53 - 0.98	0.038
Poor				0.60	0.42 - 0.86	0.005
**Distance from the village clinic**						
< 100 m				1.0		
100-500 m				1.61	1.15 - 2.26	0.006
> 500 m				1.40	0.92 - 2.12	0.113
**City**						
Liaocheng				1.0		
Binzhou				0.59	0.43 - 0.83	0.002
Zibo				0.89	0.65 - 1.24	0.494
**Awareness of FDCSs**						
Less familiar				1.0		
Know it well				11.83	7.48 - 18.72	< 0.01
**Observations**	1395					

*FDCSs* family doctor contract services

In addition, the multivariate logistic regression analysis also identified other older adults’ characteristics related to the FDCSs signing behavior. Older people with a wide variety of jobs (both agricultural and non-agricultural work) (OR = 0.49, 95% CI, 0.26 - 0.93), as well as retirees have a lower contract rate (OR = 0.43, 95% CI, 0.20 - 0.93) compared to those older people who were unemployed. Physical activity was another factor that related to the signing rate, with those who have the habit of doing physical exercise having a higher signing rate (OR = 1.34, 95% CI, 1.01 - 1.76). Older people with good self-rated health had higher contract rates than those seniors with normal (OR = 0.85, 95% CI, 0.53 - 0.98) or poor self-rated health (OR = 0.60, 95% CI, 0.42 - 0.86). Compared with the older adults who were closer to the village clinic (< 100 m), those who were further (100 - 500 m) had a higher contract rate (OR = 1.61, 95% CI, 1.15 - 2.26). Older adults who knew FDCSs well had higher signing rates than those who were less familiar with FDCSs (OR = 11.83, 95% CI, 7.48 - 18.72). The awareness rate of the rural older people in Liaocheng was higher than those in Binzhou and Zibo, and there was a significant difference between Liaocheng and Binzhou (OR = 0.59, 95% CI, 0.43 - 0.83).

## Discussion

In this study, we found that the current signing rate among the older participants was approximately 28.2%, which was consistent with previous studies focusing on the signing rate in different regions of China [14, 29]. The government target of FDCSs coverage rate was 30% in 2017, while the coverage rate of key groups contracted services reached over 60% [28]. However, the results of our study showed this goal was far from being achieved. A study from Changning district in Shanghai, China, found that there were 61.8% of residents know about FDCSs, and another study showed that the awareness rate reached 61.7% [30, 31], which were both higher than the result of our study (41.8%). We also found that 97.1% of those older adults who have not signed up were less familiar with the FDCSs. Consistent with many previous studies, our study demonstrated that the awareness of FDCSs had a strong association with the rural older adults’ contract behavior [12, 28, 32]. These findings suggest that the rural residents, especially the older adults’ awareness of FDCSs in Shandong province, should be further improved by a public information campaign.

A strong association was found between cardiovascular-metabolic multimorbidity and the higher FDCSs signing rate of the older people in rural Shandong in this study. It indicated that the rural older people with cardiovascular-metabolic multimorbidity were more likely to sign up. This could be due to that multiple chronic conditions would negatively impact the quality of life of older people [10, 33, 34]. At the same time, the number of chronic diseases and quality of life affected the utilization of healthcare services [35, 36]. The patients with chronic diseases were found to be more likely to visit a general practitioner or access to healthcare services [9, 37]. Older adults with multimorbidity preferred to using long-term care than those without multiple chronic diseases [38]. As a result, older people with cardiovascular-metabolic multimorbidity have a greater demand for healthcare services, and there would be more demand for services such as the management of chronic diseases that family doctors can provide, leading to higher signing rate.

It is worth considering that older people with a physical activity habit had a higher signing rate. To some extent, this finding indicated that this group of older adults paid more attention to their physical health and had higher demands for health monitoring and management, which is consistent with the service content of FDCSs [5, 14]. There was possible underlying reason: older people with a physical activity habit maintained better awareness of health self-management compared to those without exercise habits [39, 40], so they would be more proactive in understanding the content of FDCSs and more willing to sign up with family doctors. Older people with a physical activity habit also had more time and opportunities to talk with their neighbors in the village [41], which was an important way for them to get information about FDCSs. In the process of communication, the older adults might have better understandings of FDCSs [39, 41], thus their willingness to sign the contract might be further improved.

Distance from the village clinic was identified as a significant variable. Older adults who lived further from the village clinic (100-500 m) had higher signing rate than those lived within 100 m. That was similar with a previous research in rural US [42]. It is not difficult to imagine that the older people who lived far away from village clinics might be more willing to receive regular home visits provided by their family doctors, considering the inconvenience of their activities, thus making their contract rates of FDCSs relatively higher. Besides, there were other factors affecting the contract behaviors, such as self-rated health status, occupation, and awareness of FDCSs. Findings from previous studies were consistent with our results [32, 42–46].

The current study revealed that there was a certain gap between the signing rate among older people and its policy goal, and the older adults with cardiovascular-metabolic multimorbidity had a higher family doctor contract rate than those without cardiovascular-metabolic multimorbidity. The findings have several implications for the policy makers. Firstly, according to our findings, the awareness of FDCSs among rural older adults in Shandong province needs to be further improved. It is vital to take urgent measures to strengthen publicity and education about FDCSs, such as lectures by family doctors, mobilized by village cadres, and using mass media tools [7, 47]. Secondly, as a key group of FDCSs, the older people who suffer from chronic diseases or cardiovascular-metabolic multimorbidity were supposed to be the top priority in this policy, especially in rural areas. Primary healthcare institutions, mainly village clinics, should provide items such as regular physical examinations, follow-up management and long-term prescriptions [9, 12], to better meet the service needs of the older adults with cardiovascular-metabolic multimorbidity.

The main strength of this study is the rigorous sampling procedure, which makes our results representative. Nevertheless, there are some limitations in this study. Firstly, we only included three common chronic diseases, including hypertension, diabetes, and coronary heart disease as the reference for judging cardiovascular-metabolic multimorbidity. The results might be different if more chronic conditions would be included in this study. In the follow-up study, it will be further remedied. Secondly, it is a cross-sectional study, so it can only examine the relationship, not causation, between signing behavior and cardiovascular-metabolic multimorbidity as well as other correlative factors among rural older people. Thirdly, recall bias might exist because the main variables we used were self-reported. Fourth, conducting many tests of significance increases likelihood of type I error.

## Conclusions

The FDCSs contract rate of rural seniors in Shandong province had not achieved the preliminary target yet. Besides, several factors associated with signing behavior were found in this study, including whether the respondents had cardiovascular-metabolic multimorbidity, awareness of FDCSs, physical activities, distance from the village clinic, self-rated health status, and occupations. It is suggested that the government should make joint efforts to expand the publicity and coverage of FDCSs, give priority to meeting the health service needs of the seniors with cardiovascular-metabolic multimorbidity, as well as improve the capacity of family doctor teams.

## Data Availability

The datasets used and / or analyzed during the current study are available from the corresponding author (Prof. Chengchao Zhou) on reasonable request.

## Abbreviations

FDCSs: Family doctor contract services
FD: Family doctor
